# Phytoestrogens regulate the proliferation and expression of stem cell factors in cell lines of malignant testicular germ cell tumors

**DOI:** 10.3892/ijo.2013.2060

**Published:** 2013-08-20

**Authors:** ASTRID HASIBEDER, VIVEK VENKATARAMANI, PAUL THELEN, HEINZ-JOACHIM RADZUN, STEFAN SCHWEYER

**Affiliations:** 1 Departments of Pathology, Georg August University, D-37075 Göttingen;; 2 Hematology and Oncology, Georg August University, D-37075 Göttingen;; 3 Urology, Georg August University, D-37075 Göttingen;; 4 Pathology Starnberg, D-82319 Starnberg, Germany

**Keywords:** phytoestrogens, testicular germ cell tumors, proliferation, stem cell factors

## Abstract

Phytoestrogens have been shown to exert anti-proliferative effects on different cancer cells. In addition it could be demonstrated that inhibition of proliferation is associated with downregulation of the known stem cell factors NANOG, POU5F1 and SOX2 in tumor cells. We demonstrate the potential of *Belamcanda chinensis* extract (BCE) and tectorigenin as anticancer drugs in cell lines of malignant testicular germ cell tumor cells (TGCT) by inhibition of proliferation and regulating the expression of stem cell factors. The TGCT cell lines TCam-2 and NTera-2 were treated with BCE or tectorigenin and MTT assay was used to measure the proliferation of tumor cells. In addition, the expression of stem cell factors was analyzed by quantitative PCR and western blot analysis. Furthermore, global expression analysis was performed by microarray technique. BCE and tectorigenin inhibited proliferation and downregulated the stem cell factors NANOG and POU5F1 in TGCT cells. In addition, gene expression profiling revealed induction of genes important for the differentiation and inhibition of oncogenes. Utilizing connectivity map in an attempt to elucidate mechanism underlying BCE treatments we found highly positive association to histone deacetylase inhibitors (HDACi) amongst others. Causing no histone deacetylase inhibition, the effects of BCE on proliferation and stem cell factors may be based on histone-independent mechanisms such as direct hyperacetylation of transcription factors. Based on these findings, phytoestrogens may be useful as new agents in the treatment of TGCT.

## Introduction

Testicular cancer is the most frequently occurring malignancy in young men aged 20–39 years (1). In 2008 it was estimated that over 8,000 cases of testicular germ cell tumors (TGCT) were diagnosed in the United States and Europe, a comparably rare tumor. Furthermore, the overall incidence has increased worldwide since the turn of the century and was associated to genetic predispositions and exposure to environmental contaminants (2,3). The biology of testicular germ cell tumors is diverse, arising from a precursor lesion called intratubular germ cell neoplasia that can be found growing *in situ* within seminiferous tubules and which expresses transcription factors common to embryonic stem (ES) cells, suggesting that the cell of origin is a pluripotent gonocyte. Despite a common cell of origin, testicular cancers are histologically and clinically separated into seminoma and non-seminoma, comprising embryonal carcinoma, yolk sac tumor, choriocarcinoma and teratoma. The core stemness transcription factors POU5F1 and NANOG which are expressed in both, seminoma and non-seminoma tumor cells are thought to be pivotal for the identification of TGCT. Apart from these common markers, SOX2 has been suggested to distinguish between the two histological subtypes, expressed only in non-seminomas (4). The mammalian transcription factor POU5F1 is expressed by early embryo cells and germ cells and is essential for maintaining pluripotency (5). While lack of POU5F1 leads to apoptosis, inappropriate high expression can promote tumorigenesis (6,7). Similarly, NANOG, another transcription-factor has been described to be essential for self-renewal. Whereas NANOG disruption in ES cells results in differentiation to endoderm lineages, knockdown leads to inhibition of tumor development (8,9). A transcriptional regulatory circuitry involving the transcription factors POU5F1, SOX2, NANOG and others has been identified. Expressed specifically in pluripotent cells, they may be essential for ES cells self-renewal and differentiation. They are switched on/off by input environmental signals and they are also regulated by themselves. When these genes are expressed, the self-renewal genes are activated and the differentiated genes are repressed so ES cells can maintain their pluripotency (8). Experimental studies revealed repressive epigenetic modification in the promoter region of NANOG by histone deacetylase inhibitors (HDACi) resulting in inhibition of the transcription factors NANOG, POU5F1 and SOX2. The consequence of the knockdown of this ES-like gene signature was cell cycle arrest and differentiation in all three germ layers (10).

Phytoestrogens are of special interest in current research for different reasons. On the one hand the epidemiological incidence of malignancies is thought to be connected to the abundance of (phyto-) estrogens (11). On the other hand, the popularity in the population makes them attractive as potential drugs or supportive medicine. Studies found that e.g. postmenopausal women are more willing to take phytoestrogens instead of conventional hormone-replacement therapy describing them as ‘unnatural’ (12). The rhizome of the leopard lily *Belamcanda chinensis* is well known in traditional Chinese medicine where it is utilized to treat various symptoms and disease. Different compounds of the extract have been identified so far, including several phytoestrogens, one of the major components being tectorigenin (13). Anti-cancerogenic effects of phytoestrogens, especially of *Belamcanda chinensis* extract (BCE) and tectorigenin have been shown in diverse types of cancer and cell lines. Lee *et al* described a tumor inhibitory effect of tectorigenin in human promyelocytic leukemia HL-60 cells (14). Later, Thelen *et al* reported substantial data on the impact of tectorigenin and BCE on prostate cancer (cell lines) focusing hormone pathways with notable results (15,16).

The aim of this study was to elucidate the antitumor activity of BCE and tectorigenin on TGCT cell lines represented by TCam-2 (seminoma) cells and NTera-2 (non-seminoma). Furthermore, we attempted to elucidate the mechanism of action of this herbal drug.

## Materials and methods

### Cell culture and reagents

Human TGCT cell lines TCam-2 (seminoma) and NTera-2 (non-seminoma) were grown in RPMI-1640 (PAA Laboratories, Pasching, Austria), supplemented with 10% fetal bovine serum (PAA Laboratories), 1% penicillin/streptomycin (Invitrogen, Karlsruhe, Germany), 1% glutamine (PAA Laboratories) and 2.5% HEPES-buffer (PAA Laboratories). They were cultured in an incubator at 37°C and 5% CO_2_. After treatment for 24, 48 or 72 h cells were harvested by scraping and washed three times with PBS. Cell isolation for RNA and protein extraction was performed by centrifugation at 1,200 × g for 4 min.

The cells were treated with various concentrations of BCE (Christoffel Scientific Consulting, Buchberg-Sengenthal, Germany) or tectorigenin (Girindus, Bergisch Gladbach, Germany) solubilized in DMSO (Sigma-Aldrich Chemie, Steinheim, Germany), which was adjusted to 0.1% in all experiments inclusive the controls. Concentrations were selected according to Thelen *et al* (15,16). Valproic acid (Sigma-Aldrich Chemie) was prepared in sterile water and utilized at 5 mM. Trichostatin A (Sigma-Aldrich Chemie) 500 nM was solubilized in DMSO. Concentrations of these two HDACi were selected according to Venkataramani *et al* (17).

### Cell proliferation

Proliferation and viability of cultured cells after treatment were colorimetrically measured with an MTT assay using Cell Proliferation kit I (Roche Diagnostics, Mannheim, Germany). Therefore 4,000 cells were cultured in 100 *μ*l phenol red-free RPMI on 96-well plates and stimulated with different concentrations of BCE and tectorigenin. Further steps were carried out according to the manufacturer’s protocol.

### RNA extraction, quantification and reverse transcription-PCR

Total RNA was extracted using QIAshredder and RNeasy mini kit (Qiagen, Hilden, Germany), conducted according to the producer’s instructions. Quantity and quality were examined by a Bioanalyser 2100 utilizing the RNA 600 Nano LabChip-Kit (Agilent Technologies, Waldbronn, Germany). Reverse transcription-PCR of 500 ng total cellular RNA with Random hexamer primers was done using Omniscript RT kit (Qiagen).

### Quantitative real-time PCR (qRT-PCR)

To analyze RNA expression, PCRs were run by using gene-specific primers. To investigate the stem cell factors, PCR amplification was performed using specific primer sets (Eurofins MWG Operon, Ebersberg, Germany) for NANOG (upstream primer, 5′-TTCCTTCCTCCATGGATCTG; downstream primer, 5′-ATCTGCTGGAGGCTGAGGTA), POU5F1 (upstream primer, 5′-AGAAGGATGTGGTCCGAGTG; downstream primer, 5′-GTGAAGTGAGGGCTCCCATA) and SOX2 (upstream primer, 5′-CAAGATGCACAACTCGGAGA; downstream primer, 5′-CTCCGGGAAGCGTGTACTTA). A specific primer set for ARP (upstream primer, 5′-CGACCTGGAAGTCCAACTAC; downstream primer, 5′-ATCTGCTGCATCTGCTTG) was used as a control. Each sample was composed of 10 *μ*l qPCR MasterMix Plus for SYBR-Green I w/fluorescein (Eurogentec, Cologne, Germany), 0.15 *μ*l downstream and 0.15 *μ*l upstream primer and 4.7 *μ*l RNase-free water. Individual PCR programs were designed and amplification and fluorescence measurements were made with iCycler iQ Real-Time PCR Detection System (Bio-Rad, Munich, Germany). The achieved data were analyzed using appropriate software (Bio-Rad).

### Microarray analysis

RNA was extracted using the TRIzol method. Microarrays were done using the Low RNA Input linear Amplification Kit Plus, One Color protocol (Agilent Technologies, Waldbronn, Germany). RNA was labeled (mono-color experiment) and hybridized to the *C. elegans* 4×44K design array from Agilent Technologies (Waldbronn, Germany). Quantity and Cy-dye incorporation rates of the generated target material were measured using a NanoDrop ND-100. Washing and staining of the arrays were done according to the manufacturer’s recommendation. Cy3 intensities were detected by one-color scanning using an Agilent DNA microarray scanner (G2505B) at 5 micron resolution. Scanned image files were visually inspected for artifacts and then analyzed. Intensity data were extracted using Agilent’s Feature Extraction (FE) software, version 9.5 and analyzed using the Limma package of Bioconductor (18,19). To find over-represented functions we used DAVID (http://david.abcc.ncifcrf.gov/13.04.2010).

### Connectivity map (cmap)

The UniGene IDs were translated into Affymetrix IDs. Querying the connectivity map was performed by using version build 02 (http://www.broadinstitute.org/cmap/13.08.2011).

### Nuclear protein extraction and quantification

Nuclear lysates were gained by using NucBuster Protein Extraction Kit (EMD Biosciences, Madison, WI, USA). The quantification was performed after the well-established method according to Bradford, utilizing ready-to-use Roti-Quant Kit (Roth, Karlsruhe, Germany).

### Western blot analysis

After preparing appropriate protein concentration of 25 *μ*g, SDS-PAGE was performed using 4–12% Vario-Gels (Anamed-Elektrophorese GmbH, Gross-Bieberau, Germany). Separation of proteins by electrophoresis was followed by the transfer to nitrocellulose membranes (GE Healthcare Europe GmbH, Munich, Germany) and afterwards blocked for 1 h with 10% non-fat milk in TBS-T. Primary antibodies were diluted in TBS-T/5% BSA and incubated with membranes overnight at 4°C. The following primary antibodies were used: NANOG, POU5F1, histone H3 (Santa Cruz Biotechnology Inc., Santa Cruz, CA, USA), SOX2 (Abcam plc, Cambridge, UK), acetyl-histone H4 (Upstate Millipore, Billerica, MA, USA) and β-actin (Sigma-Aldrich Corporation, St. Louis, MO, USA). The membranes were washed 3X with TBS-T before the secondary antibody (Dako Denmark A/S, Glostrup, Denmark) was added. Proteins were visualized on X-ray film (Hyperfilm EC, Amersham Biosciences, Freiburg, Germany), scanned and analyzed using ImageJ software (version 1.41o, National Institute of Health).

## Results

### BCE and tectorigenin inhibit proliferation of TGCT cell lines

**Figure 1.**
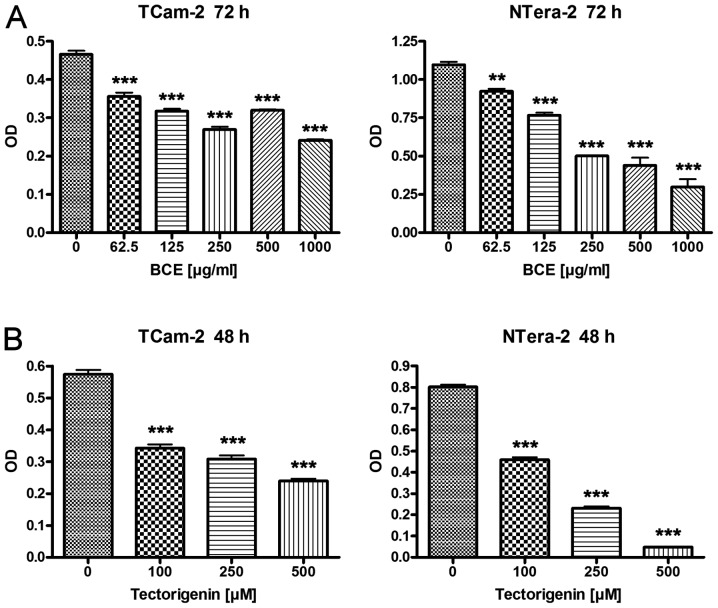
Proliferation of TGCT cell lines after treatment with BCE and tectorigenin. TCam-2 and NTera-2 cells were treated with various concentrations of phytoestrogens for 72 and 48 h, respectively. Proliferation was measured with an MTT assay. Both cell lines showed concentration-dependent reduced viability after treatment with (A) BCE as well as (B) tectorigenin. OD = optical density. Statistics (t-test), ^**^p<0.01; ^***^p<0.001.

To evaluate the effects of BCE and tectorigenin on proliferation the TGCT cell lines TCam-2 and NTera-2 were stimulated with 62.5, 125, 250, 500 and 1,000 *μ*g/ml of BCE for 24 and 72 h, or 100, 250 and 500 *μ*M of tectorigenin for 24 and 48 h, respectively. After 24 h stimulation with different concentrations of BCE and tectorigenin cell lines TCam-2 and NTera-2 showed no or minimal differences in the proliferation rate compared to the controls, respectively (data not shown). In contrast TCam-2 and NTera-2 showed a significant reduction of proliferation in a dosage-dependent manner for tectorigenin after 48 h and BCE after 72 h, respectively (Fig. 1). Based on the morphological observation that changes typical for apoptosis or cytotoxicity could not be detected in the TGCT cell lines the results are suggestive for changes in gene expression.

### Differential expression of stem cell factors in TGCT cell lines after phytoestrogen treatment

Various stem cell genes are important for self-renewal and are also associated with poorly differentiated tumors (20). Three important stem cell genes the NANOG, POU5F1 and SOX2 are normally enriched in embryonic stem cells. Based on these findings we investigated the expression of these stem cell factors in TCGT cell lines after stimulation with BCE.

After stimulation with various concentrations of BCE (62.5, 125, 250, 500 or 1,000 *μ*g/ml) for 24 and 72 h, tumor cells were analyzed by qRT-PCR or western blot analyses for the expression of the stem cell genes NANOG, POU5F1 and SOX2, respectively.

**Figure 2.**
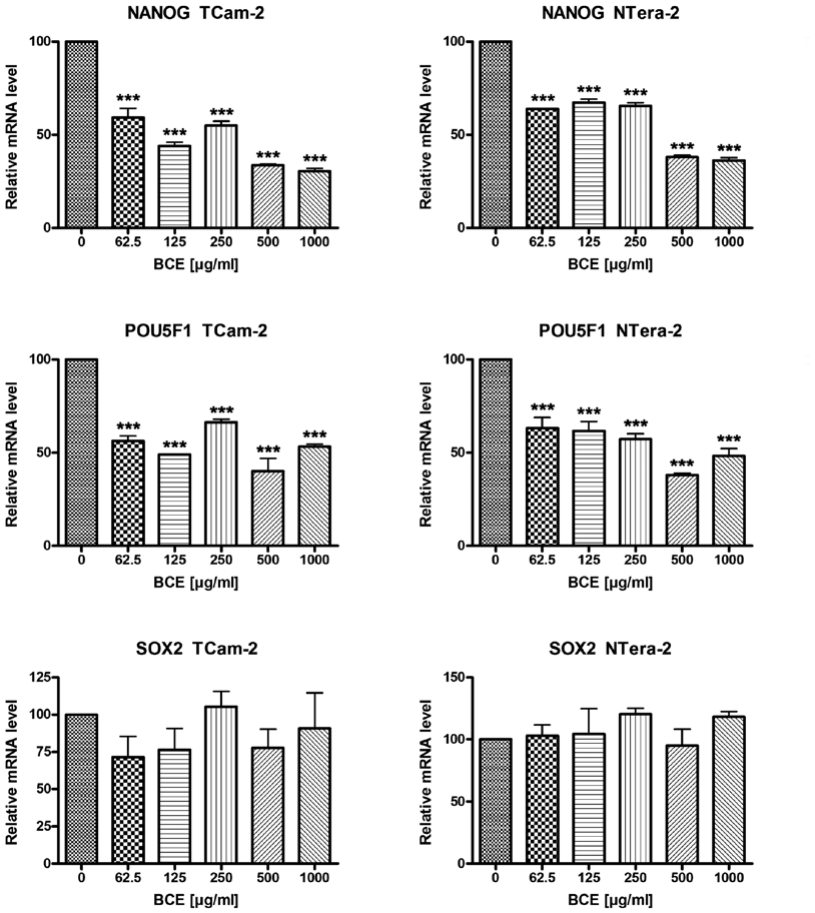
mRNA expression of stem cell factors in TGCT cell lines after BCE treatment. TCam-2 and NTera-2 cells were treated with various concentrations of BCE for 24 h and relative mRNA expression was measured with qRT-PCR. Both cell lines showed reduced expression of the stem cell factors NANOG and POU5F1, whereas SOX2 showed no alteration. Statistics (t-test), ^***^p<0.001.

**Figure 3.**
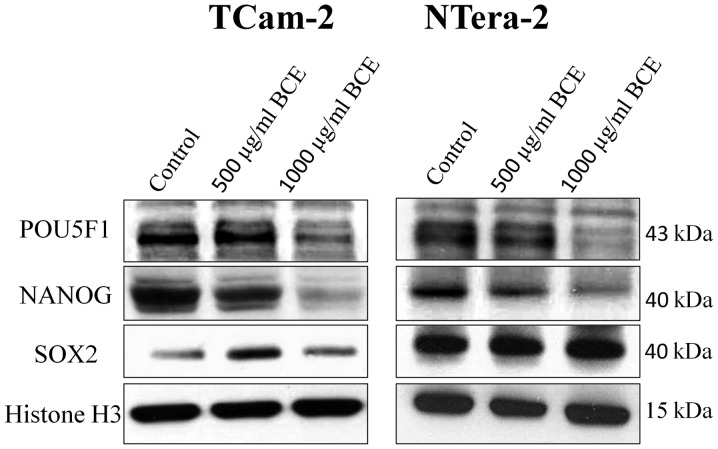
Protein expression of stem cell factors in TGCT cell lines after BCE treatment. TCam-2 and NTera-2 cells were treated with various concentrations of BCE for 24 h and protein expression was measured with western blot analysis. Both cell lines showed reduced expression of the stem cell factors NANOG and POU5F1, whereas SOX2 showed no alteration. Histone H3 served as control.

As shown in Fig. 2, BCE stimulation caused a significant decrease of NANOG and POU5F1 mRNA expression in a dosage-dependent manner in the TGCT cell lines TCam-2 and NTera-2, respectively. Depending on BCE concentration NANOG mRNA expression is reduced up to 70% in TCam-2 and 64% in NTera-2, respectively. In contrast, mRNA expression of SOX2 remained unchanged in both tumor cell lines. Consequently, we asked whether BCE treatment of TGCT cell lines for 24 h alters the protein expression of the stem cell genes. Western blot analyses revealed that according to mRNA expression NANOG and POU5F1 proteins were significantly inhibited in a dosage-dependent manner. In concordance with the mRNA expression, protein expression of SOX2 showed no different expression as compared to the control (Fig. 3).

### Phytoestrogen-induced gene expression profiling in TGCT cell lines

Gene expression profiling using microarray analysis was performed after treatment of both TGCT cell lines TCam-2 and NTera-2 with 1,000 *μ*g/ml BCE for 72 h. Different numbers of genes were up- or downregulated in TCam-2 and NTera-2 cells, respectively. Further analyses were focused on differential expression of genes important for differentiation as well as carcinogenesis and proliferation. The results showed that genes important for differentiation (e.g. β-catenin, AP-2γ) are induced whereas genes being involved in carcinogenesis and proliferation (e.g. phospholipase A2, GDF-3) are inhibited (Table I and II).

### An attempt to identify the mode of action of BCE on TGCT cell lines utilizing cmap

Connectivity map (cmap) is a reference collection of gene-expression profiles from cultured human cells treated with bioactive small molecules. The resource tries to provide a systematic approach to discover functional connections for example among diseases, drug action or any small molecules sharing a mechanism of action (21).

Gene profiles of BCE-stimulated TCam-2 and NTera-2 cells were gained from microarray analysis (see above). Querying the cmap revealed different substances causing similar and contrary gene profiles in seminoma and non-semi-noma cell lines. High connections were achieved with HDACi for both TGCT types (NTera-2>TCam-2) but also with other different kind of drugs (Table III and IV). Interestingly, estrogens and antagonists, as well as genistein (as a phytoestrogen being represented in the cmap) showed very low connection to BCE-induced profiles, indicating different mechanism of action (data not shown).

### Comparing effects of HDAC inhibitors with BCE

**Figure 4.**
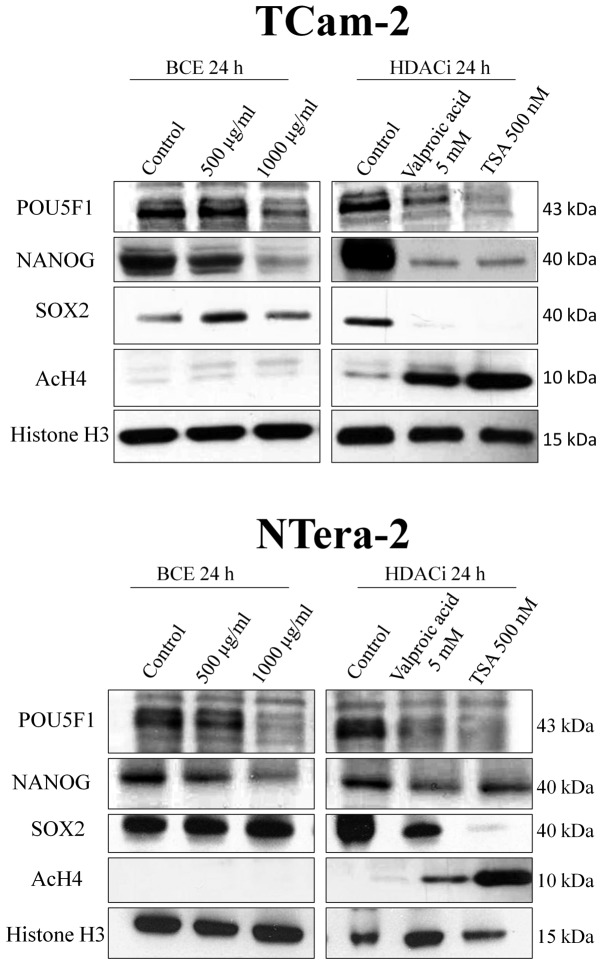
Comparison of the effects of HDAC inhibitors and BCE. TGCT cell lines were treated with various concentrations of BCE and with two HDAC inhibitors (HDACi) for 24 h. Protein expression was measured with western blot analysis. Stimulation of both TCam-2 and NTera-2 cells with HDAC inhibitors valproic acid and trichostatin A (TSA) lead to a decrease of protein expression of the stem cell genes NANOG, POU5F1 and SOX2 and to hyperacetylation of histone protein H4. In contrast, BCE inhibits only NANOG and POU5F1 protein expression and causes no acetylation of histone protein H4 in the cell lines. Histone H3 served as control.

Based on our results of cmap and the published data of the depletion of the embryonic stem cell signature by histone deacetylase inhibitors (10), we investigated the effect of BCE on histone deacetylase inhibition. We demonstrated that stimulation of both TGCT cell lines with HDAC inhibitors valproic acid and trichostatin A (TSA) leads to a significant decrease of protein expression of the stem cell genes and the decrease is accompanied by a hyperacetylation of histone protein H4. In contrast, the phytoestrogen-induced inhibition of NANOG and POU5F1 protein expression is independent of acetylation of histone protein H4 (Fig. 4).

## Discussion

In this study, we showed that BCE and tectorigenin inhibit the proliferation of TGCT cells in a time- and concentration-dependent manner. The anti-proliferative potential of BCE and the isolated isoflavone thereof tectorigenin has been evaluated on different cancer types and cell lines (14,22). Showing time-and concentration-dependent effects on proliferation in human promyelocytic leukemia HL-60 cells, Lee *et al* also described induction of differentiation in these cells. They reasoned that the anti-proliferative effect of tectorigenin was ascribed to induction of differentiation and apoptosis (14). Recent studies refuted this because reduction of cells was exclusively associated with inhibition of proliferation (changes in G1, S and G2M phase) but not with increasing amount of apoptosis. The conclusion was that the inhibitory effect on cell viability is based on cell cycle arrest (22).

At first sight tectorigenin seems more capable in reduction of cell viability than BCE. Focusing the used concentrations of BCE (62.5–1,000 *μ*g/ml) and tectorigenin (100–500 *μ*M) shows that a direct comparison is not feasible. Morrissey *et al* described that 100 *μ*g/ml BCE comprise 17 *μ*M tectorigenin (22) and Thelen *et al* also specified the content of tectorigenin in BCE of about 5%. BCE and tectorigenin were directly compared before with consistent results (15). Later a potential synergistic effect from the combination of phytochemicals in BCE was hypothesized because tumor cell proliferation and androgen receptor expression were more affected by BCE than by the pure isoflavone tectorigenin alone (16). But there are also different views for the variable potential of tectorigenin and BCE. For example the 5-hydroxyl group of the isoflavone structure of several phytoestrogens plays a leading role for cytotoxic effects (14). Equally to other phytoestrogens tectorigenin comprises this structure (22,23). BCE also includes other compounds containing this structure as well as isoflavone glycoside (25) which is poorly permeable for cell membranes (14).

Furthermore, we investigated whether treatment of TGCT cells with phytoestrogens influence the expression of stem cell factors involved in self-renewal and proliferation of poorly differentiated tumors (10,20). BCE is capable of downregulating the expression of the stem cell factors NANOG and POU5F1 in TGCT cells, whereas expression of SOX2 remained unchanged. The detection of SOX2 in seminoma cells TCam-2 is inconsistent with other authors (26), though it was described later that this cell line also features non-seminomatous characteristics (4). Previous studies on HDACi-treated stem cells and embryonal carcinoma cells showed a connection between downregulation of NANOG and inhibition of proliferation, however, the exact relation between cell cycle arrest and NANOG suppression is not yet known. Furthermore You *et al* described dependency of the transcription factors POU5F1 and SOX2 from NANOG in NANOG-siRNA experiments and stimulation with high dose of apicidin. The conclusion was that additionally to the known circuitries, other mechanisms are in involved in the downregulation of POU5F1 and SOX2, independent of NANOG (10). Previous studies exhibited incoherent effects on the dependency of these three stem cell factors (27), being later interpreted as incomplete knockdown (10). Also, results from estrogen-related receptor β-knockdown showed that NANOG is depleted followed by reduction of POU5F1 expression ascribed as POU5F1-dependent inhibition of NANOG (28). However, further investigations are needed to explore the circuitries as well as additional factors.

Effects of phytoestrogens on stem cell factors have been described before by analyzing genistein. The isoflavonoid genistein contained in soy induces downregulation of NANOG and decreased protein levels of POU5F1 and NANOG. It was concluded that the observed decrease in the transcript level of NANOG is a downstream effect of genistein-induced depletion of POU5F1 protein (29). Furthermore it should be noted, that in our experiments NANOG and POU5F1 mRNA expression are just mildly repressed compared with the protein expression where both stem cell factors were barely detectable after stimulation with BCE and tectorigenin, respectively. One explanation may be that protein analyses do not provide quantitative results, or that other mechanisms such as post-transcriptional gene silencing are involved.

Gene expression analyses of TCam-2 and NTera-2 cells revealed several genes differentially expressed after BCE treatment. Using DAVID we focused on genes involved in differentiation or associated with malignancies. Genes with repressed expression in stimulated NTera-2 cells: GDF3, which is also expressed in primordial germ cells, is overexpressed in TGCT compared with normal testis, just as NANOG and POU5F1 (30). DAZL is only expressed in IGCNU but not for example in breast cancer cells and is therefore regarded as germ cell origin of these cells (30). CALCA stimulates growth and motility of prostate cancer cells and also has essential functions in angiogenesis (31). In addition EGFL6 abets, probably through paracrine mechanisms, angiogenesis and promotes migration of endothelia cells (32). GLI1-knockout experiments showed that suppression of this transcription factor compromises proliferation, invasion and migration of cancer cells (33). Similar effects are known for ASCL2. Liver metastases from colorectal cancer exhibit an ASCL2-related stem cell signature which likely influences the metastatic activity of tumor cells (34).

Induced genes in stimulated NTera-2 cells: HAND1 is known to be involved in morphogenesis and embryogenesis (35). Additionally it is induced in GADD45G-overexpressed NCCIT cells, as well as in POU5F1-knockout cells and therefore is involved in cell cycle arrest and differentiation (36). Besides being involved in morphogenesis (37) HMX2, like HAND1, is repressed in cancer and induction seems to be involved in inhibition of proliferation (38).

Repressively expressed genes in stimulated TCam-2 cells: GDF3, CALCA and EGFL6 are inhibited as well in TCam-2 as in NTera-2 cells. For information on their function in differentiation see above. Furthermore, AKT1-expression was reduced, being known for central roles in proliferation and survival pathways in cancer (39). Similar mechanisms have been reported for ANGPTL4, which supports tumor progression through metastasis and vasculogenesis (40).

Induced gene expression in stimulated TCam-2 cells: MAFB induces differentiation (41), coexistently it is known as oncogene in multiple myeloma (42). Altogether the maf protein can play antagonistic functions in oncogenesis and plays a dual role as oncogene and tumor suppressor-like protein (43). NEUROG3 has important functions in specialization of organs (44,45). GADD45B expression is associated with the level of differentiation of tumors being clearly expressed at lower levels in poorly differentiated compared to well differentiated tumors. Hence it was proposed as a marker for the state of differentiation of tumors (46). In summary, BCE-stimulated TGCT cell lines revealed repression of oncogenes and induction of genes central for differentiation indicating anti-cancerogenic activities of this substance.

Assuming a connection between the repression of NANOG and POU5F1 and hyperacetylation of histone proteins by genistein (29,47,48) we investigated whether BCE acts similarly by also affecting hyperacetylation. HDACi showed, comparable with BCE/tectorigenin, inhibition of the stem cell signature and inhibition of proliferation, probably in relation with cell cycle arrest (10). No hyperacetylation of histone H4 by BCE highlights the different mode of action of phytoestrogens despite the comparable effects on the stem cell signature through these two agents.

Attempting to identify the mode of action of BCE on TGCT cell lines we utilized cmap. BCE-induced gene signature in NTera-2 cells revealed high connections to the HDAC inhibitors vorinostat, CP-690334-01 and trichostatin A. Given that BCE does not cause hyperacetylation of histone H4 we assume that the observed commonalities are based on histone-independent mechanisms like direct hyperacetylation of various transcription factors (49). Genistein, showing almost no congruence to BCE, seems to act in a different way and that the observed similarities are just exceptions. Additionally it should be noted that we used high dose of BCE compared to genistein in the cmap (1,000 *μ*g/ml BCE for 72 h vs. 10 *μ*M genistein) presumably provoking toxic effects.

TCam-2 cells showed just one high ranged HDACi, CP-690334-01. Strongest positive connection was obtained for lisuride, which is used in treatment of prolactinoma. Beside inhibition of prolactin secretion it is also known for reduction of tumor cell mass and inhibition of transcription (50), furthermore necrotic effects have been detected (51). By viewing the ‘permuted results’, genistein had hardly any connection to BCE in TCam-2 cells. In contrast, on the ‘detailed results’ table BCE revealed the two highest connections with genistein (used concentrations see above).

Overall BCE acts differently in seminoma and non-semi-noma cells. To seize again the suggestion that mechanisms similar to HDACi may be involved, it has been described that the effects are partially dependent of the cell type (49) and that different target structures of HDACi are differentially expressed in TGCT. For example the DNA methyltransferase DNMT1 is not expressed in seminoma, however, in embryonal carcinoma it is induced (52).

In summary, we demonstrated that the phytoestrogens BCE and tectorigenin are capable of inhibiting the proliferation of TGCT cell lines and lead to a downregulation of the stem cell genes NANOG and POU5F1. Furthermore, various kinds of genes involved in differentiation and carcinogenesis were differentially regulated by phytoestrogens in TGCT cells. In addition cmap reveals high positive connections to histone deacetylase inhibitors (HDACi) but BCE stimulation had no effect on histone deacetylase inhibition thus histone-independent mechanisms such as direct hyperacetylation of transcription factors are possible. Further investigations are needed to clarify the molecular mechanism(s) of phytoestrogens being beneficial in the treatment of TGCT.

## Figures and Tables

**Table I.** Phytoestrogen-induced gene expression profiling in TCam-2 cells.

Symbol	Description	Stimulated vs. control
TDGF1	Teratocarcinoma-derived growth factor 1	−3.6
GDF3	Growth differentiation factor 3	−3.3
AICDA	Activation-induced cytidine deaminase	−3.1
MAL2	Mal, T-cell differentiation protein 2	−2.9
NANOG	Nanog homeobox	−2.8
CALCA	Calcitonin-related polypeptide alpha	−2.8
SFRP2	Secreted frizzled-related protein 2	−2.7
GDF15	Growth differentiation factor 15	−2.7
SLC7A5	Solute carrier family 7, member 5	−2.4
AKT1	V-akt murine thymoma viral oncogene homolog 1	−2.4
TNP1	Transition protein 1	−2.3
DIAPH2	Diaphanous homolog 2	−2.3
ANGPTL4	Angiopoietin-like 4	−2.2
PLP1	Proteolipid protein 1	−2.2
EGFL6	EGF-like-domain, multiple 6	−2.1
IRX3	Iroquois homeobox 3	2.0
SOX3	SRY(sex determining region Y)-box 3	2.0
EGR2	Early growth response 2	2.0
TFAP2C	Transcription factor AP-2γ	2.1
MYH9	Myosin, heavy chain 9, non-muscle	2.1
MAFB	V-maf musculoaponeurotic fibrosarcoma oncogene homolog B	2.2
DHRS2	Dehydrogenase/reductase (SDR family) member 2	2.2
NEUROG3	Neurogenin 3	2.2
HAND1	Heart and neural crest derivatives expressed 1	2.2
GADD45B	Growth arrest and DNA-damage-inducible, beta	2.2
FOXC1	Forkhead box C1	2.2
JAG1	Jagged 1	2.3
ID3	Inhibitor of DNA binding 3, dominant negative helix-loop-helix protein	2.3
AXIN2	Axin 2	2.3
SEMA4D	Sema domain (semaphorin) 4D	2.4
NEUROG2	Neurogenin 2	2.5
ZIC2	Zic family member 2	2.6
HES1	Hairy and enhancer of split 1	2.6
NEUROG1	Neurogenin 1	2.7
TOB1	Transducer of ERBB2, 1	3.0
CTNNB1	Catenin (cadherin-associated protein), beta 1	3.2

Gene expression profiling using microarray analysis was performed after treatment of the TGCT cell line with 1,000 *μ*g/ml BCE for 72 h. Analysis was focused on differential expression of genes important for differentiation as well as carcinogenesis and proliferation. Genes important for differentiation were induced whereas genes being involved in carcinogenesis and proliferation were inhibited.

**Table II.** Phytoestrogen-induced gene expression profiling in NTera-2 cells.

Symbol	Description	Stimulated vs. control
PLA2GA	Phospholipase A2, group IIA	−4.5
DAZL	Deleted in azoospermia-like	−4.2
GDF3	Growth differentiation factor 3	−4.1
GPNMB	Glycoprotein (transmembrane) nmb	−3.5
DAZ2	Deleted in azoospermia 2	−3.1
CALCA	Calcitonin-related polypeptide alpha	−3.1
GDF15	Growth differentiation factor 15	−2.9
TDGF1	Teratocarcinoma-derived growth factor 1	−2.7
GLI1	GLI family zinc finger 1	−2.6
DMRTB1	DMRT-like family B with proline-rich C-terminal, 1	−2.5
ASCL2	Achaete-scute complex homolog 2	−2.4
AICDA	Activation-induced cytidine deaminase	−2.3
LPL	Lipoprotein lipase	−2.2
THRB	Thyroid hormone receptor, beta	−2.1
EGFL6	EGF-like-domain, multiple 6	−2.1
HAND1	Heart and neural crest derivatives expressed 1	2.6
HMX2	H6 family homeobox 2	2.7

Gene expression profiling using microarray analysis was performed after treatment of the TGCT cell line with 1,000 *μ*g/ml BCE for 72 h. Analysis was focused on differential expression of genes important for differentiation as well as carcinogenesis and proliferation. Genes important for differentiation were induced whereas genes being involved in carcinogenesis and proliferation were inhibited.

**Table III.** Results of the cmap query for connections of BCE-stimulated TCam-2 cells with gene signatures induced by other substances viewing the ‘permuted results’ table.

Rank	Cmap name	n	Mean	P-value
1	Meticrane	5	−0.719	0.00004
2	Lisruride	5	0.624	0.00006
3	Medrysone	6	−0.725	0.00006
4	Doxorubicin	3	−0.819	0.00026
5	Vigabatrin	3	0.689	0.00036
6	H-7	4	−0.670	0.00060
7	0173570-0000	6	−0.585	0.00068
8	CP-690334-01	8	0.437	0.00076

Gene expression profiling of the TGCT cell line was carried out by using microarray analysis after treatment of the cells with 1,000 *μ*g/ml BCE for 72 h.

**Table IV.** Results of the cmap query for connections of BCE-stimulated NTera-2 cells with gene signatures induced by other substances viewing the ‘permuted results’ table.

Rank	Cmap name	n	Mean	P-value
1	Vorinostat	12	0.592	0.00000
2	CP-690334-01	8	0.545	0.00000
3	Trichostatin A	182	0.394	0.00000
4	Securinine	4	−0.588	0.00016
5	Monensin	6	0.557	0.00032
6	Perphenazine	5	0.444	0.00050

Gene expression profiling of the TGCT cell line was carried out by using microarray analysis after treatment of the cells with 1,000 *μ*g/ml BCE for 72 h.
